# Epigenetic Study in Parkinson’s Disease: A Pilot Analysis of DNA Methylation in Candidate Genes in Brain

**DOI:** 10.3390/cells7100150

**Published:** 2018-09-26

**Authors:** Luis Navarro-Sánchez, Beatriz Águeda-Gómez, Silvia Aparicio, Jordi Pérez-Tur

**Affiliations:** 1 Unitat de Genètica Molecular, Instituto de Biomedicina de Valencia, CSIC, 46010 València, Spain; sannalu84@hotmail.com (L.N.-S.); beatrizaguedagomez@gmail.com (B.Á.-G.); silapdo@gmail.com (S.A.); 2 Centro de Investigación Biomédica en Red en Enfermedades Neurodegenerativas (CIBERNED), 46010 València, Spain; 3 Unidad Mixta de Genética y Neurología, Instituto de Investigación Sanitaria La Fe, 46026 València, Spain

**Keywords:** epigenetics, Parkinson’s disease, brain, DNA methylation

## Abstract

Efforts have been made to understand the pathophysiology of Parkinson’s disease (PD). A significant number of studies have focused on genetics, despite the fact that the described pathogenic mutations have been observed only in around 10% of patients; this observation supports the fact that PD is a multifactorial disorder. Lately, differences in miRNA expression, histone modification, and DNA methylation levels have been described, highlighting the importance of epigenetic factors in PD etiology. Taking all this into consideration, we hypothesized that an alteration in the level of methylation in PD-related genes could be related to disease pathogenesis, possibly due to alterations in gene expression. After analysing promoter regions of five PD-related genes in three brain regions by pyrosequencing, we observed some differences in DNA methylation levels (hypo and hypermethylation) in *substantia nigra* in some CpG dinucleotides that, possibly through an alteration in Sp1 binding, could alter their expression.

## 1. Introduction

Parkinson’s disease (PD) is a progressive neurodegenerative disorder characterized by neural loss in *substantia nigra pars compacta* and the presence of numerous Lewy bodies in surviving neurons [1]. Affected individuals suffer from postural instability, tremor at rest, bradykinesia, and rigidity, as well as other clinical signs such as secondary motor symptoms and non-motor symptoms involving abnormalities in cognition/behavior/sensory system, sleep disorders, and autonomic dysfunctions [2].

Despite being the second most common neurodegenerative disorder, with a prevalence of 4–5% by the age of 85 [3], the mechanism that triggers the neurodegenerative process is still unknown, as is whether the biological processes which are altered in PD (oxidative stress, mitochondrial dysfunction, protein aggregation, inflammation, altered Ca^2+^ homeostasis) are causes or consequences.

Pathogenic mutations have been described only in around 10% of patients, mainly in monogenic or familial forms of PD [4,5,6,7]. They are found in 6 genes: *SNCA* [8], *PRKN* [9], *PINK1* [10], *DJ-1* [11], *LRRK2* [12], and *VSP35* [13]. Nevertheless, the remaining 90% of PD patients suffer from idiopathic or sporadic forms of PD with unknown cause. According to this, PD is considered a prototypic multifactorial, or complex, disorder.

Recently, some studies have also focused on epigenetics: mitotically and/or meiotically heritable changes that cannot be explained by changes in DNA sequence, i.e., stable and long-term alterations not present in the DNA sequence [14].

Epigenetics include RNA-mediated processes, histone modifications, and DNA methylation. They are flexible and dynamic processes that change depending on time and environment. Therefore, one person carries one genome, but hundreds or even thousands of epigenomes that could explain different phenotypes [15].

Differences in miRNA expression, histone modifications, and DNA methylation levels have been described in PD patients. With regard to this last epigenetic mechanism, no differences in DNA methylation levels in the brain have been observed between patients and controls in *UCHL1*, *MAPT* promoter, and *PRKN* promoter [16,17] whereas contradictory results have been reported for CpG islands in intron 1 of *SNCA* [18,19,20]. In PD patients and animal models, it has been demonstrated that α-synuclein could sequester DNMT1, which maintains DNA methylation, in the cytoplasm leading to global DNA hypomethylation [21]. These results highlight the possible influence that DNA methylation could have on PD origin by regulating genes linked to PD. Although more studies are needed to confirm this hypothesis, these reports point out that not only genetic variability should be considered in the future when looking for pathogenic factors in Parkinson’s disease.

Singleton et al. first described a triplication in *SNCA* causing familial PD [22], pointing towards the fact that an increase in the expression of this protein may have the same consequences as a pathological mutation. As expression levels can also be regulated by altering DNA methylation in the promoter region, we wanted to analyze the possible relation between epigenetics and PD, more specifically, whether DNA methylation levels were altered in the brain of PD cases, and thus, would affect their expression levels. We focused on 5 extensively studied genes (*SNCA*, *PRKN*, *PINK1*, *DJ-1* and *LRRK2*) which are responsible for familial PD.

## 2. Material and Methods

### 2.1. Subjects

Frozen *substantia nigra*, parietal cortex, and occipital cortex were obtained from the Biobanc HCB-IDIBAPS, Barcelona (Catalonia, Spain) for 5 controls and 5 PD patients. All subjects gave their informed consent for inclusion before participating in the study. The study was conducted in accordance with the Declaration of Helsinki, and the protocol was approved by the Institutional Review Board from the Biobanc. Their clinical status was confirmed by *post-mortem* brain analysis. Controls, unlike PD patients, did not present Lewy bodies. However, 4 had other neurological injuries such as vascular encephalopathy and/or AD related pathology; in addition, 3 of them had suffered from vascular dementia.

There were 3 men and 2 women in each group. The mean age at the moment of death for the controls was 77.80 ± 6.80 years, whereas for the PD cases, was 81.00 ± 3.81 years. The mean post-mortem delay was 6 h 2 min for the controls and 10 h 14 min for the PD cases.

### 2.2. DNA Extraction

We followed the Maxwell 16 Mouse Tail DNA Purification Kit (Promega, Madison, WI, USA) instructions to extract DNA from each individual brain region: around 30 mg of tissue were dissected and then introduced into the Maxwell 16 Instrument (Promega, Madison, WI, USA). Finally, the sample was quantified using the Qubit dsDNA BR Assay kit and the Qubit Fluorometer (Life Technologies, Carlsbad, CA, USA).

For one of the controls, we could not obtain DNA from *substantia nigra*, so for this brain region our results are based on the values of 4 individuals in the control group.

### 2.3. Bisulfite Treatment

We treated the DNA with bisulfite, as in this process unmethylated C converts into U, whereas 5 mC remains unaltered. It is the gold standard technique to analyze DNA methylation levels. We opted for the EZ DNA Methylation-Gold kit (Zymo Research, Irvine, CA, USA) to treat 1 μg of DNA per individual brain region, following the supplier instructions. Finally, it was quantified using NanoDrop 2000 (Thermo Scientific, Waltham, MA, USA), taking into account that bisulfite-treated DNA has an absorption coefficient at 260 nm, resembling that of RNA.

### 2.4. CpG Island Prediction

We focused on the 5 most extensively studied genes in PD. All of them are responsible for familial forms of Parkinson’s disease: *SNCA*, *PRKN*, *PINK1*, *DJ-1,* and *LRRK2*. As their expression is ubiquitous, although with some differences between regions, CpG islands in their promoters should exhibit low methylation levels. Therefore, gene transcription should not be repressed, thus allowing their expression in all the cells [23,24].

In order to determine the location of the CpG island, for each gene we considered around 3000 bp upstream and 3500 bp downstream from the transcription start site (TSS) to include the promoter and the first exons, where CpG islands are frequently present. We predicted the presence of CpG islands using 5 prediction programs: Softberry (http://www.softberry.com/berry.phtml?topic=cpgfinder&group=programs&subgroup=promoter), CpG cluster (http://bioinfo2.ugr.es/CpGcluster/) [25], Bioinformatics (http://www.bioinformatics.org/sms2/cpg_islands.html) and Emboss (http://www.ebi.ac.uk/Tools/seqstats/emboss_cpgplot/). Furthermore, we annotated the CpG islands predicted at the UCSC genome browser [26]. The CpG cluster is based on the physical distance between neighboring CGs, and not in the search of CpG islands [27] as others are; therefore, it can find shorter CpG islands. Also, we predicted the position of putative promoters for those genes using FirstEF (http://rulai.cshl.edu/tools/FirstEF/) and WWW Promoter Scan (http://www-bimas.cit.nih.gov/molbio/proscan/).

As results differed between predictions, we chose for our analysis those areas where all or most of the predictions agreed (Figure S1, Supplementary Material).

### 2.5. Primer Design and Pyrosequencing

The methylation analysis was carried out by pyrosequencing. Ten pyrosequencing assays were designed using the PyroMark Assay Design Software 2.0 (Qiagen, Hilden, Germany), avoiding homopolymers longer than 4 residues, with a maximum difference in primers T_m_ of 2 °C, an amplicon length between 100–500 bp, no more than two CG dinucleotides in the primer sequence, and with some T bases derived from unmethylated C to ensure only amplification of bisulfite modified DNA [28]. In the target sequences, we analyzed the presence of frequent SNPs using the Single Nucleotide Polymorphism database, dbSNP (http://www.ncbi.nlm.nih.gov/projects/SNP/), because their presence could alter the assay. There was none in any of the regions analyzed by our assays.

In the Supplementary Material, Table S1, all the pyrosequencing assays conducted are described, as are the primer sequences, the size of the fragment amplified, and the PCR conditions.

For each assay we performed three independent replicates. All pyrosequencing reactions were carried out in a PyroMark MD sequencer using NDTS (nucleotide dispensing tips) at the Servei d’Anàlisi d’ADN of the Instituto de Biomedicina de Valencia-CSIC. All reagents were from Qiagen. Results were analyzed by the program PyroMark Q-CpG 1.0.9, which calculated the percentage of DNA methylation per position.

Furthermore, we checked for PCR bias [29]. For this purpose, PCRs were carried out with DNA with known methylation percentages (0, 50 and 100) which were previously bisulfite-treated. Then, DNA methylation levels were analyzed by pyrosequencing. We confirmed that the observed and the expected methylation levels matched. There was an efficiency of conversion of unmethylated cytosines into uracils of nearly 100%, and, thus there was no bias. We used the EpiTect PCR Control DNA Set from Qiagen.

### 2.6. Statistical Analysis

Due to the low number of samples, a non-parametric test (the Mann-Whitney test) was used to compare means. The statistical analysis was conducted using SPSS (IBM Analytics, Armonk, NY, USA), version 20.

Results are shown as “overall”, representing the arithmetic mean of the methylation levels at all CpG dinucleotides in an assay, as well as for the individual CpG dinucleotides. The arithmetic mean is based on the values obtained for the 5 individuals that compose each group (controls and PD patients), except for the results from the control group in *substantia nigra*, which are based on only 4 individuals, as we could not obtain DNA for one of the controls.

### 2.7. Transcription Factor Binding Site Prediction

We predicted in silico whether CpG dinucleotides differentially methylated could be affecting transcription factor binding sites using TFSEARCH (http://www.cbrc.jp/research/db/TFSEARCH.html) [30], JASPAR CORE (http://jaspar.genereg.net/) [31] and AliBaBa 2.1 and PATCH (http://www.gene-regulation.com/pub/programs.html). The expression of each transcription factor in brain was taken from The Human Protein Atlas (http://www.proteinatlas.org) [32] and the Allen Human Brain Atlas (2010), from The Allen Institute for Brain Science (http://human.brain-map.org) [33].

## 3. Results

In Table 1, we summarize the significant results of those CpG dinucleotides differentially methylated in patients compared to controls. The results obtained for individual CpG dinucleotides, and overall for the 10 pyrosequencing assays, are shown in Figures S2–S6 (Supplementary Material).

No single island showed differences in its level of methylation when the information for all CpG dinucleotides was combined (Supplementary Figures S2–S6), although some specific positions in *SNCA*, *PRKN* and *PINK1* showed differences between cases and controls in *substantia nigra*. Also, some significant differences were observed in individual dinucleotides in both parietal and occipital cortices.

For these sites, we checked if they could be part of transcription factor binding sites, and thus, whether changes in their methylation level could affect gene expression.

Some candidates were proposed by sequence-based prediction programs, but due to their expression pattern that did not include *substantia nigra*, and/or their targets that did not include our genes, only Sp1 could be considered as a possible candidate; it is ubiquitous and binds to GC-rich sequences. Moreover, it is involved in many cellular processes, including cell differentiation, cell growth, apoptosis, immune responses, response to DNA damage, and chromatin remodeling. It can be an activator or a repressor, and its activity is highly regulated by post-translational modifications. It interacts with HDAC1 and DNMT1 [34,35,36], amongst others. The precise location of the Sp1 binding sites can be found in Table 1 and supplementary Figure S1. Most of the Sp1 putative binding sites are found within the predicted promoters, also affecting in some instances (*SNCA* #2, *PRKN* #2 and *PINK1* #1) exonic and intronic sequences that are included in the prediction.

## 4. Discussion

DNA methylation has been implicated in a diverse range of cellular functions and pathologies. It is generally associated with a repressed chromatin state and inhibition of promoter activity, i.e., transcriptional repression [37,38]. We analyzed whether the five genes responsible for the familial forms of Parkinson’s disease could be related to PD pathogenesis by having differential DNA methylation patterns. Our focus was on DNA methylation levels around their transcription start site, because variations of this epigenetic mark in this area can influence gene expression.

Overall, no major differences were observed, although for a few positions, the level of methylation of specific CpG dinucleotides differed significantly between cases and controls. Nevertheless, these changes stem from a very low level of methylation, i.e., small variations in the methylation level could have a large numerical impact. Due to those two characteristics, no statistically significant results would have been obtained if corrections for multiple tests were applied.

Most statistically significant islands share one common, and suggestive, characteristic: Sp1 is the only transcription factor that could be mediating the effect of the differential methylation seen here. Nevertheless, more work is needed to confirm our results prior to exploring the role of Sp1 in PD.

For *SNCA*, overexpression is pathogenic [22], and thus, lower DNA methylation levels that involve higher transcription and gene expression could be pathogenic too. However, for *PRKN*, *PINK1*, *DJ-1,* and *LRRK2*, the pathogenic factor in PD is the lack of enough active protein; therefore, higher DNA methylation levels that involve transcription silencing could lead to decreased gene expression, and thus, could be pathogenic [9,10,11,12]. As expected for housekeeping genes, the promoters of *SNCA*, *PRKN*, *PINK1*, *DJ-1,* and *LRRK2* were poorly methylated in all regions analyzed, allowing their ubiquitous expression. Our few statistically significant results were obtained for specific CpG sites mainly located in *SNCA* and *PRKN*. The majority of them showed that DNA methylation levels were higher in controls than in cases in those specific CpG dinucleotides. Remarkably, a higher methylation level was observed only for two specific CpG dinucleotides in *SNCA* with respect to controls (Table 1).

We analyzed *substantia nigra* and parietal and occipital cortices, with a special focus on the first region, as it is extensively affected in PD [39], unlike the parietal cortex and occipital cortex, that could be considered “control brain regions”. We did not compare the values between brain regions because, as Ladd-Acosta et al. concluded, the DNA methylation pattern correlates much more strongly within a brain region across individuals than within an individual across brain regions [40]. Nevertheless, we found CpG sites with statistically significant differences in all three tissues (Table 1), and in some instances with the same CpG dinucleotide being altered in more than one region.

Previous studies also addressed the question of whether methylation levels in PD-related genes could be linked to disease pathogenesis, although with controversial results possibly related to, in part, methodological and design differences and to study limitations. In those studies, when statistical significance is found, the results point towards a hypomethylation in PD cases.

In brain, with regard to *SNCA*, Matsumoto et al., Jowaed et al. and de Boni et al. analyzed, in a similar number of individuals to our study, a region in *SNCA* that was not included in our study, although close and downstream to the area in intron 1 that we studied [18,19,20]. Matsumoto et al. [18] and Jowaed et al. [19] reported hypomethylation across *substantia nigra* in PD cases when compared to controls (and Jowaed et al. even in cortex and *putamen* [19]), whereas de Boni et al. [20] didn’t observe any difference in any of the five brain regions analyzed, *substantia nigra* included, and not even for global values on the region analyzed or single CpG dinucleotides. Our results are similar to those of de Boni et al. [20], although we could see some hypo and hypermethylation in some specific CpG dinucleotides. In addition, our technical procedure is similar, using pyrosequencing instead of cloning and sequencing, as Matsumoto et al. and Jowaed et al. did, which could result in a technically-biased evaluation [18,19]. We all analyzed *substantia nigra* from PD cases and controls, despite the fact that dopaminergic neurons are largely eliminated in this region in PD cases as a consequence of the evolution of the disease, and are almost intact in healthy subjects. Nevertheless, the range of our values was similar to those observed by Jowaed et al. and de Boni et al., i.e., very low levels, which would not be affected by such cellular heterogeneity [19,20]. In the opposite direction, the methylation levels observed by Matsumoto et al. were completely different, and this difference could be related to their methodological approach [18].

For *PRKN*, de Mena et al. observed that there was almost no methylation in any of the 3 brain regions analyzed in cases and controls in the region of the analyzed promoter, which does not overlap with our assays [17]. There are similarities between that study and ours, in terms of the number of individuals and regions analyzed (we studied parietal cortex instead of cerebellum); although, again, we used pyrosequencing instead of cloning and sequencing. This could explain the different results observed between that study and ours. We reported some differences in specific CpG dinucleotides for this gene (Table 1).

In addition, our methodological approach does not differentiate between 5-hydroxy-methylC (5 hmC) and 5-methylC. Although 5 hmC has its highest levels in the central nervous system, and its amount increases with age, Munzel et al. and Kriaucionis et al. observed that this effect on methylation percentages is expected to be almost imperceptible [41,42].

Although there are no studies that have analyzed the influence of the post-mortem delay on DNA methylation, previous reports did not find any effect on pH, RNA quality [43], or protein concentration (post-synaptic proteins [44] and primary microglia [45]) in brain. Therefore, we considered that the difference in post-mortem delay between both groups, healthy controls, and PD cases would not affect our results.

Masliah et al. concluded, after a genome-wide study of methylation levels in brain and blood, that there were similar methylation patterns between them [46]. Unfortunately, no blood was available from the individuals we have analyzed here, so we could not test the hypothesis that epigenetic marks could be potential biomarkers for non-invasive predictive testing for PD. In blood, some studies have been carried out where no differences between cases and controls for SNCA [47,48] or for *PRKN*, *DJ-1* [49,50] were observed. However hypomethylation has been described in other studies for sporadic cases [51,52,53]. Tan et al. also observed no differences in other regions of *SNCA* and *LRRK2*, both overlapping with our assays [53]. The main difference between these studies relies on the number of individuals analyzed, as different technical approaches have given similar results.

We conclude that imbalances in the methylation of specific CpG islands in PD-related genes do not seem to be related to the disease process. We studied three different tissues in five different genes in triplicates, which increases the confidence of our results. However, due to the small size of our population and the low level of methylation observed, our results could be considered as trends that should be replicated in a larger study, maybe using genome-wide approaches to account for the effect of genes not related to familial PD. Moreover, functional validation of these results is also needed. This is especially significant, as doubling or halving the amount of methylation with such a low baseline is unlikely to have a major impact on the pathophysiology of the disease.

Other epigenetic mechanisms, i.e., small RNAs or histone modification, could also be relevant in the dysregulation of the expression of some genes important in the pathogenesis of Parkinson’s disease. For example, miR-7 [54] and miR-153 [55] repress α-synuclein expression, and miR-205 expression is down regulated in sporadic PD patients with enhanced LRRK2 expression [56]. In addition, it has been reported that the expression of some miRNAs is altered in in vivo PD models [57,58] and in PD patients [59]. On the other hand, it has also been observed that α-synuclein associates with histones and inhibits their acetylation [60]. Therefore, histone deacetylase (HDAC) inhibitors are the most recent emerging therapeutic targets in the treatment of PD. It has been demonstrated in cellular and animal models that the neurotoxicity of α-synuclein in the nucleus could be rescued by their administration [61,62]. Thus, it is important to study the epigenetic regulation of PD genes in order to obtain a clearer picture of the etiology of this disorder.

## Figures and Tables

**Table 1:** Statistically significant results for the comparison of DNA methylation levels between controls and PD patients (*p* < 0.05).

Assay ^1^	Position ^2^	Region ^3^	Location ^4^	*p*-Value	Methylation Ratio ^5^	Sp1 Binding ^6^
*SNCA* #1	3	PC	−1586	0.008	2.571	NB
*SNCA* #1	6	PC	−1551	0.016	2.958	−strand
*SNCA* #1	6	SN	−1551	0.016	0.000	−strand
*SNCA* #2	2	OC	−1458	0.032	0.326	NB
*SNCA* #2	7	OC	−1442	0.008	0.526	+strand
*SNCA* #2	8	SN	−1440	0.016	0.033	+strand
*PRKN* #1	3	SN	−187	0.016	0.193	NB
*PRKN* #2	2	OC	+44	0.032	0.114	+strand
*PRKN* #2	6	PC	+69	0.032	0.128	+strand
*PINK1* #1	2	SN	+355	0.016	0.143	+strand

^1^: Each assay is named with the gene followed by the number of the fragment analyzed, according to notation in Figure S1 and Table S1, supplementary material. ^2^: Position indicates the CpG pair within the assay, as shown in supplementary Figures S2–S6. ^3^: PC: parietal cortex; OC: occipital cortex; SN: substantia nigra. ^4^: +1 was assigned to the A from the first translated codon. The position is taken using the following references: NM_001146054 (SNCA), NM_013988 (PRKN) and NM_032409 (PINK1). ^5^: Ratio obtained by dividing the methylation level in PD patients with respect to its equivalent in healthy individuals. ^6^: When a Sp1 site is predicted to exist in the region where there is a CpG pair showing statistical significant differences between cases and controls, the strand where this binding may exist is shown. NB: not binding predicted for Sp1 nor any other transcription factor.

## Supplementary Materials

The following are available online at http://www.mdpi.com/2073-4409/7/10/150/s1, Figure S1: Schematic representation of the regions of interest in SNCA (A, B), LRRK2 (C, D), PRKN (E, F), PINK1 (G, H) and DJ-1 (I, J), Figure S2: Results for all three assays in SNCA in the parietal and occipital cortices and Substantia nigra from PD patients and controls, Figure S3: Results for the two assays in LRRK2 in the parietal and occipital cortices and Substantia nigra from PD patients and controls, Figure S4: Results for the two assays in PRKN in the parietal and occipital cortices and Substantia nigra from PD patients and controls, Figure S5: Results for the assay in PINK1 in the parietal and occipital cortices and Substantia nigra from PD patients and controls, Figure S6: Results for the two assays in DJ-1 in the parietal and occipital cortices and Substantia nigra from PD patients and controls, Table S1. Description of the parameters used for the epigenetic analysis of DNA methylation levels by pyrosequencing, Table S2. Characteristics of the CpG islands predicted by the software employed in this work.
