# Supplementary material for: Epigenetic Study in Parkinson’s Disease: A Pilot Analysis of DNA Methylation in Candidate Genes in Brain
Source: Cells. 2018 Sep 26;7(10):150. doi: 10.3390/cells7100150 (PMC6210421; doi:10.3390/cells7100150)
Supplement: Supplementary file 1 [file cells-07-00150-s001.pdf]

Table S1. Description of the parameters used for the epigenetic analysis of DNA methylation levels by pyrosequencing.

Gene	Assay	Primers sequence (5´→ 3´)	Amplicon (bp)	CpGs analyzed	Number of PCR cycles (X)	Annealing temperature (Y; ºC)	Target
SNCA	1	F: GGGGAAAGAGGAAGAGG**T**<br>R: *CCCTCTCTT**AAA**CCCCTTCT**A**<br>Pyroseq (F): GGAG**T**A_**A**_G**T**TG**T**AGGGAAAG**T**A	340	6	45	63	Predicted promoter
SNCA	2	F: AGG**T**AGG_**A**_GG**T**TGGAGTTGAT<br>R: *T**AA**CCACTCCCA**A**TTCTCC<br>Pyroseq (F): GGG**TTT**AAGAGAGGGGG	380	8	38	61	Predicted promoter
SNCA	3	F: GGAGAA**T**TGGGAGTGG**TT**AT<br>R: *CACA**AA**TACTTACCT**A**A**A**TCCCTCT**A**C<br>Pyroseq (F): GGGTTTG**TT**T**TTT**A**T**T**TTTTT**AG	262	5	45	60	Exon 1
LRRK2	1	F: GGGG**T**T**T**_**A**_GGG**TT**TGTGGA**T**<br>R: *TCCCTCTCC**_C_A**A**A**CCCTCCT**A**C<br>Pyroseq (F): AG**TT**AGG**TT**_**A**_GG**T**T**TT**AG**T**AG**T**	307	9	45	65	Predicted promoter
LRRK2	2	F: **TT**TGAGTGGGGGAGGAGGAA<br>R: ***A**CCACTA**A**CCAT**AA**T**AA**CACCT**A**CTTC<br>Pyroseq (F): AG**T**TG**TT**T**TT**TT**TT**T**T**ATAAA**T**AGG	254	9	45	63	Exon 1
PRKN	1	F: AGAG**T**TG**T**AA**T**AAG**T**TT**TT**AAAGGTAAG**T**<br>R: *CTCCCACCA**A**C**_C_A**CTCTCCT**AAA**TTA<br>Pyroseq (F): GGGGGG**T**TGGGGG**T**A	284	4	45	60	Predicted promoter
PRKN	2	F: GATAGGTA_**A**_GTGGGTA**TT**TG**TT**AGGTA**T**AG<br>R: *ACTTT**AA**CCCC_**C**_TCATT**A**ACA**A**TT**AA**CACC<br>Pyroseq (F): A**TT**TG**TT**AGGTA**T**AG**TT**T**T**T**T**TG	124	9	38	58	Predicted promoter partially overlapping with intron 1
PINK1	1	F: TGGTG_**A**_GGG**TT**TGGGG**T**TG<br>R: ***A**CCCC_**C**_CTCACCT**AA**ATCTCCT**A**AC<br>Pyroseq (F): **T**TGGG**TT**T**T**AT_**A**_GAGGAAAAA**T**AG	142	5	38	61	Predicted promoter overlapping with exon 1
DJ-1	1	F: GGGAGG**TT**TGGA**TT**AGAGT**TT**<br>R: *ACCCCCCAC_**C**_AAT**AA**CACA**A**TCC<br>Pyroseq (F): GG**TT**TGGA**TT**AGAGT**TT**TAA**T**AG	229	6	38	61	Predicted promoter
DJ-1	2	F: GGTGG_**A**_GGTAGAGA**T**TGTTAAG**TTT**<br>R: *CACCCCACACCA**AA**CT**A**A<br>Pyroseq (F): TGTGGGG**T**TGAGGGA	273	8	45	60	Predicted promoter overlapping with exon 1

*All the reverse primers were biotinylated in 5´.

T (in forward primers) and **A** (in reverse primers) denote the converted unmethylated cytosines whereas ***A*** (in forward primers) and ***C*** (in reverse primers) correspond to cytosines in CpG dinucleotides and are thus introduced as mismatches to overpass those variable positions.

PCR conditions: 95°C 15´; X cycles (94°C 30", Y^{o}C 30", 72°C 30"); 72°C 10´; 4°C ∞

PCR mix per one reaction (1X) for a final volume of 25µL: 17.25µL Milli-Q water + 2.5µL 10X buffer + 1µL dNTPs 5mM each + 2.5µL $MgCl_2$ 25mM + 0.5µL primerF 10 µM + 0.5µL primerR 10 µM + 0.25µL Maxima Hot Start *Taq* DNA polymerase (Thermo Scientific) 5U/µL + 0.5µL bisulfite treated DNA 50ng/µL.

Table S2. Characteristics of the CpG islands predicted by the software employed in this work. In this table, for each gene and for each program, we show the length (in bp) of the predicted CpG islands as well as their GC content. When a particular software predicted several CpG islands, they are shown separated by “,” (comma)

Gene	Program	CpG island length (bp)	% GC content
SNCA	Bioinformatics	1761	60
	CpG cluster	282, 579, 149, 306	60, 69, 61, 58
	UCSC	862	67
	Emboss	591	69
	Softberry	364	71
LRRK2	Bioinformatics	899	66
	CpG cluster	649	72
	UCSC	558	76
	Emboss	403, 235	73, 74
	Softberry	282	78
PRKN	Bioinformatics	1027	67
	CpG cluster	778	72
	UCSC	641	73
	Emboss	772	72
	Softberry	522	75
PINK1	Bioinformatics	969	67
	CpG cluster	779	73
	UCSC	506	75
	Emboss	749	74
	Softberry	435	80
DJ-1	Bioinformatics	1075	63
	CpG cluster	840	68
	UCSC	925	66
	Emboss	335, 507	63, 70
	Softberry	925	66

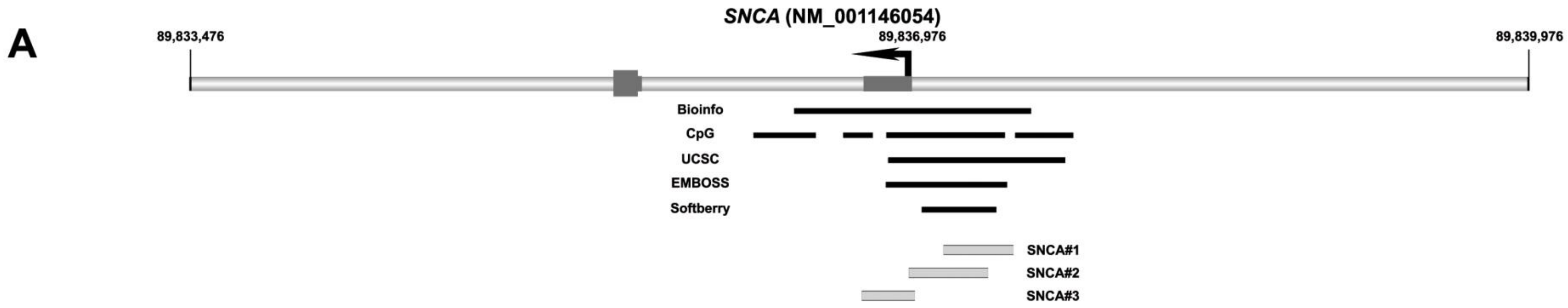

B

SNCA#1

```
GGGGAAAGAGGAAGAGGCATCATCCCTAGCCCAACCGCTCCCGATCTCCACAAGAGTGCTCGTGACCCTA
AACTTAACGTGAGGCGCAAAAGCGCCCCCACTTTCCCGCCTTGCGCGGCCAGGCAGGCGGCTGGAGTTGA
TGGCTCACCCCGCGCCCCCTGCCCCATCCCCATCCGAGATAGGGACGAGGAGCACGCTGCAGGGAAAGCA
GCGAGCGCCGGGAGAGGGGCGGGCAGAAGCGCTGACAAATCAGCGGTGGGGGCGGAGAGCCGAGGAGAAG
GAGAAGGAGGAGGACTAGGAGGAGGAGGACGGCGACGACCAGAAGGGGCCCAAGAGAG
```

SNCA#2

```
AGGCAGGCGGCTGGAGTTGATGGCTCACCCCGCGCCCCCTGCCCCATCCCCATCCGAGATAGGGACGAGG
AGCACGCTGCAGGGAAAGCAGCGAGCGCCGGGAGAGGGGCGGGCAGAAGCGCTGACAAATCAGCGGTGGG
GGCGGAGAGCCGAGGAGAAGGAGAAGGAGGAGGACTAGGAGGAGGAGGACGGCGACGACCAGAAGGGGCC
CAAGAGAGGGGGCGAGCGACCGAGCGCCGCGACGCGGAAGTGAGGTGCGTGCGGGCTGCAGCGCAGACCC
CGGCCCGGCCCCTCCGAGAGCGTCCTGGGCGCTCCCTCACGCCTTGCCTTCAAGCCTTCTGCCTTTCCAC
CCTCGTGAGCGGAGAACTGGGAGTGGCCA
```

SNCA#3

```
GGAGAACTGGGAGTGGCCATTCGACGACAGGTTAGCGGGTTTGCCTCCCACTCCCCCAGCCTCGCGTCGC
CGGCTCACAGCGGCCTCCTCTGGGGACAGTCCCCCCCGGGTGCCGCCTCCGCCCTTCCTGTGCGCTCCTT
TTCCTTCTTCTTTCCTATTAAATATTATTTGGGAATTGTTTAAATTTTTTTTTTAAAAAAAGAGAGAGGC
GGGGAGGAGTCGGAGTTGTGGAGAAGCAGAGGGACTCAGGTAAGTACCTGTG
```

Figure S1. Schematic representation of the regions of interest in *SNCA* (A, B), *LRRK2* (C, D), *PRKN* (E, F), *PINK1* (G, H) and *DJ-1* (I, J). (A, C, E, G, I). Representation of the region analyzed using the bioinformatic tools mentioned in the Materials and Methods section. Narrow dark grey boxes represent non-coding exons; wider dark grey boxes represent coding exons. Sense of transcription is shown by a black arrow on top of the region. Black bars represent the islands identified by the different web servers. Grey bars represent the assays designed in this study with their respective name on the right. **(B, D, F, H, J)** DNA sequence of the assays in A, C, E, G, I respectively. In grey, the sequencing primer used in the pyrosequencing reactions. Black boxes represent the CpG dinucleotides analyzed in this study.

C

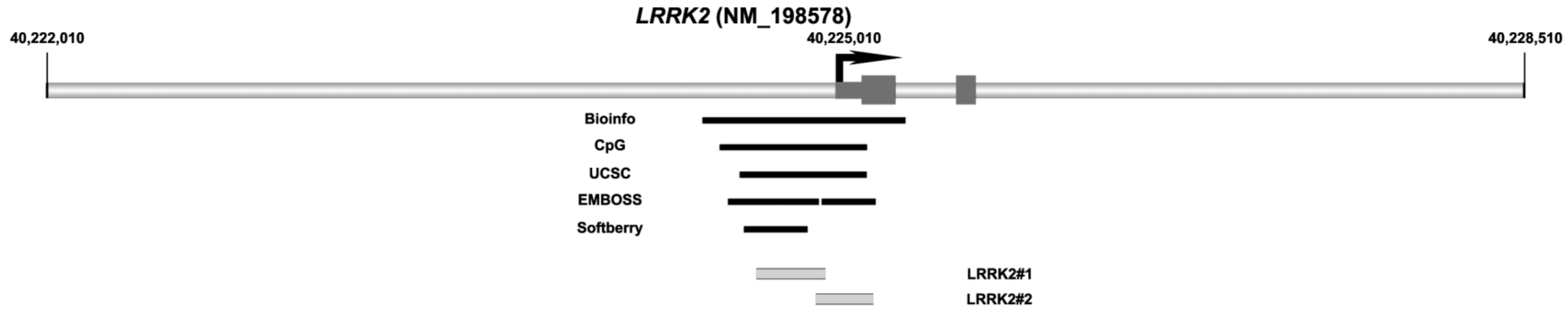

D

LRRK2#1
```
GGGGCTCCGGGCCTGTGGACTCAGCGGAGTCCGCTGAGTCAGTTTCTTCCCGCGCGACTCCCGGCCGCGC
CGCCGCTGCGGTGGAATCTGGTCCCAGGAGGCGGCGTCCGCCCGGGGTCCGGTCTAGGCGTGCGTGGGGG
CCACGGTCACGGTCATCCCAGCCAGGCCCGGCTCCAGCAGCCCCACGGCCGCCGCCAGAGTTCTGCGCGG
CCCGTCGCCTCGGCGGAGCCTCTGGCAGGCCCCTGAGCTCGTTTTTGGGGCCTGAGTGGGGGAGGAGGAA
GCCGAGCAGGAGGGCTCCGGAGAGGGA
```

LRRK2#2
```
CCTGAGTGGGGGAGGAGGAAGCCGAGCAGGAGGGCTCCGGAGAGGGAGGGCAACGCGGGGCGGGGAGCTG
CCTCCTTCCTCATAAACAGGCGGGCGTGGGCGCCGATGGGGCCCGCGGGGAGCGCTGGCTGCGGGCGGTG
AGCTGAGCTCGCCCCCGGGGAGCTGTGGCCGGCGCCCCTGCCGGTTCCCTGAGCAGCGGACGTTCATGCT
GGGAGGGCGGCGGGTTGGAAGCAGGTGCCACCATGGCTAGTGGC
```

Figure S1 (cont). Schematic representation of the regions of interest in *SNCA* (A, B), *LRRK2* (C, D), *PRKN* (E, F), *PINK1* (G, H) and *DJ-1* (I, J). (A, C, E, G, I). Representation of the region analyzed using the bioinformatic tools mentioned in the Materials and Methods section. Narrow dark grey boxes represent non-coding exons; wider dark grey boxes represent coding exons. Sense of transcription is shown by a black arrow on top of the region. Black bars represent the islands identified by the different web servers. Grey bars represent the assays designed in this study with their respective name on the right. **(B, D, F, H, J)** DNA sequence of the assays in A, C, E, G, I respectively. In grey, the sequencing primer used in the pyrosequencing reactions. Black boxes represent the CpG dinucleotides analyzed in this study.

E

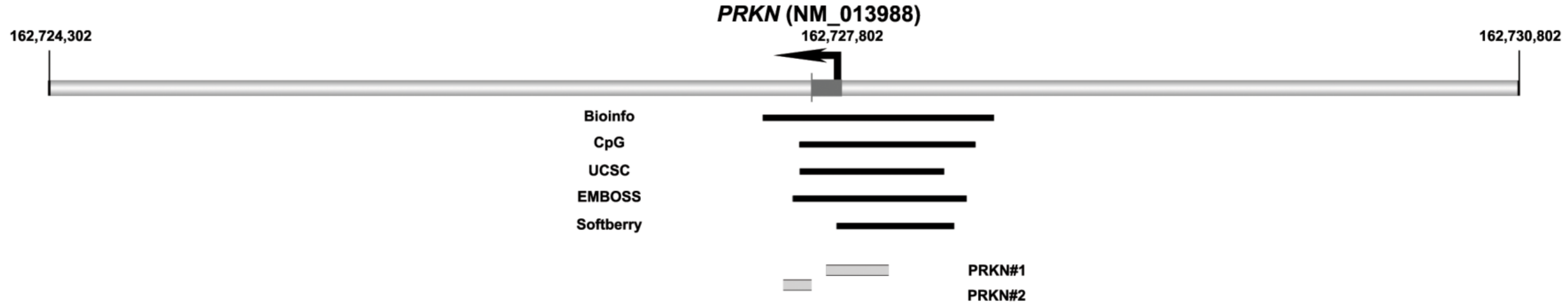

F

PRKN#1
TGCAACAAGCTTCCAAAGGTAAGCCTCCCGGTTGCTAAGCGACTGGTCAACACGGCGGGCGCATAGCCCC
GCCCCCCGGTGACGTAAGATTGCTGGGCCTGAAGCCGGAAAGGGCGGCGGTGGGGGCTGGGGGCAGGAG
GCGTGAGGAGAAACTACGCGTTAGAACTACGACTCCCAGCAGGCCCTGGGCCGCGCCCTCCGCGCGTGCG
CATTCCTAGGGCCGGGCGCGGGGGCGGGGAGGCCTGGAGGATTTAACCCAGGAGAGCCGCTGGTGGGAG

PRKN#2
GATAGGTACGTGGGTACCTGCCAGGTACAGCCTCTCTGCGCCGCCCCACGCCCCGGTATGGCGCCACGCC
CCGCCTCGGCCGACCAGGACCCCCGGTGCCAACTGTCAATGACGGGGCCAAAGT

Figure S1 (cont). Schematic representation of the regions of interest in *SNCA* (A, B), *LRRK2* (C, D), *PRKN* (E, F), *PINK1* (G, H) and *DJ-1* (I, J). (A, C, E, G, I). Representation of the region analyzed using the bioinformatic tools mentioned in the Materials and Methods section. Narrow dark grey boxes represent non-coding exons; wider dark grey boxes represent coding exons. Sense of transcription is shown by a black arrow on top of the region. Black bars represent the islands identified by the different web servers. Grey bars represent the assays designed in this study with their respective name on the right. **(B, D, F, H, J)** DNA sequence of the assays in A, C, E, G, I respectively. In grey, the sequencing primer used in the pyrosequencing reactions. Black boxes represent the CpG dinucleotides analyzed in this study.

G

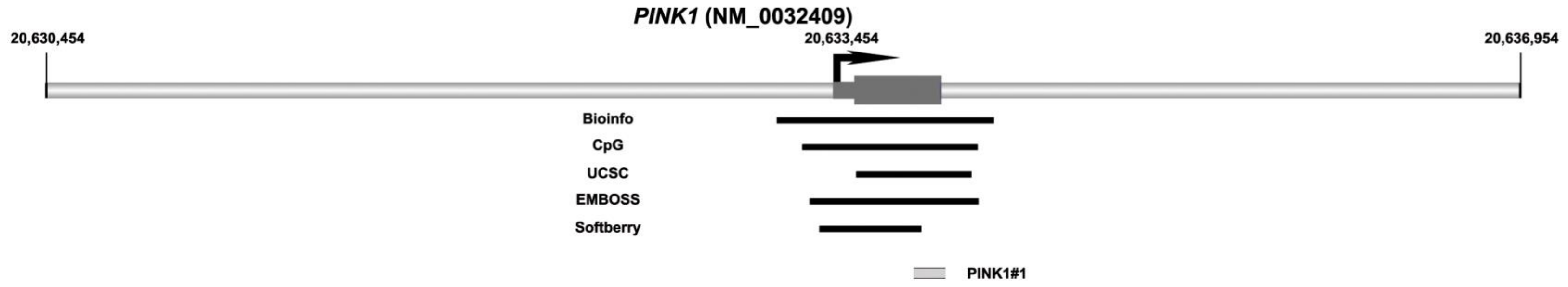

H

PINK1#1 TGGTGCGGGCCTGGGGCTGCGCGGGCCCTTGCGGCCGGGCAGTCTTTCTGGCCTTCGGGCTAGGGCTGGG
CCTCATCGAGGAAAAACAGGCGGAGAGCCGGCGGGCGGTCTCGGCCTGTCAGGAGATCCAGGTGAGCGGG
GC

Figure S1 (cont). Schematic representation of the regions of interest in *SNCA* (A, B), *LRRK2* (C, D), *PRKN* (E, F), *PINK1* (G, H) and *DJ-1* (I, J). (A, C, E, G, I). Representation of the region analyzed using the bioinformatic tools mentioned in the Materials and Methods section. Narrow dark grey boxes represent non-coding exons; wider dark grey boxes represent coding exons. Sense of transcription is shown by a black arrow on top of the region. Black bars represent the islands identified by the different web servers. Grey bars represent the assays designed in this study with their respective name on the right. **(B, D, F, H, J)** DNA sequence of the assays in A, C, E, G, I respectively. In grey, the sequencing primer used in the pyrosequencing reactions. Black boxes represent the CpG dinucleotides analyzed in this study.

I

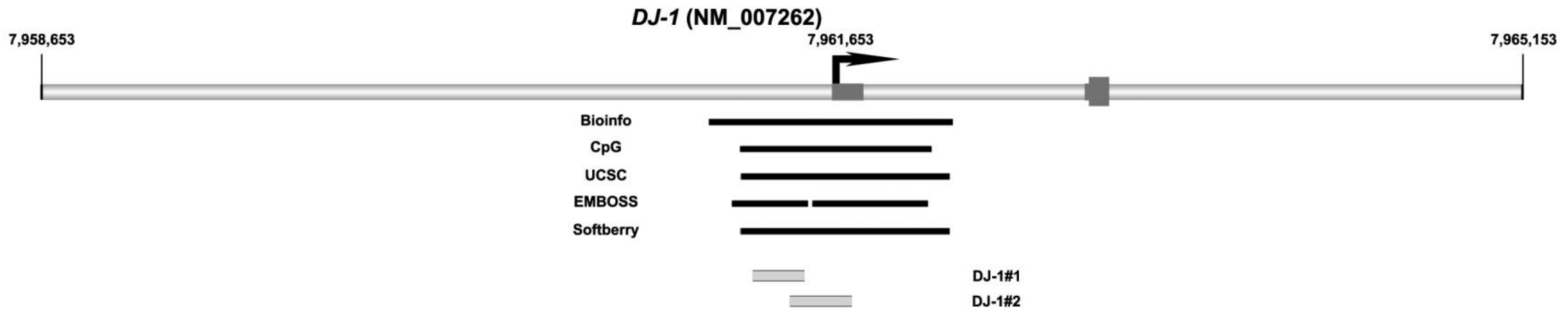

J

DJ-1#1
GGGAGGCCTGGACCAGAGTCCTAACAGACCAGAGGCGAAACGGGAAGGCGCGCCAGAAAAGGAACAACGC
AAAGGGAGCAGGCGTGCACGGAGCGCGAACTAAGGAACCCCTCTGACAACCCCAGTCCCTCGGCAGTTCC
AGAGACCGGCTCCTCACGGAGGGTGGCGGTAGAGACTGTTAAGCCCCGCGGGCGCCGGGGCAGGCCGGAC
TGTGCCATTCGTGGGGGGT

DJ-1#2
GGTGGCGGTAGAGACTGTTAAGCCCCGCGGGCGCCGGGGCAGGCCGGACTGTGCCATTCGTGGGGGGTAC
CATGTGGGACCGAGCCGCCTCACCCAGGGCTGTCCAGCTAGAAACTCCCCGGTGCCACCCCCGCCTCAGT
CCGAGGTAGACTCGGCCGGACGTGACGCAGCGTGAGGCCAAGGCGGCGTGAGTCTGCGCAGTGTGGGGCT
GAGGGAGGCCGGACGGCGCGCGTGCGTGCTGGCGTGCGTTCATTTTCAGCCTGGTGTGGGGTG

Figure S1 (cont). Schematic representation of the regions of interest in *SNCA* (A, B), *LRRK2* (C, D), *PRKN* (E, F), *PINK1* (G, H) and *DJ-1* (I, J). (A, C, E, G, I). Representation of the region analyzed using the bioinformatic tools mentioned in the Materials and Methods section. Narrow dark grey boxes represent non-coding exons; wider dark grey boxes represent coding exons. Sense of transcription is shown by a black arrow on top of the region. Black bars represent the islands identified by the different web servers. Grey bars represent the assays designed in this study with their respective name on the right. **(B, D, F, H, J)** DNA sequence of the assays in A, C, E, G, I respectively. In grey, the sequencing primer used in the pyrosequencing reactions. Black boxes represent the CpG dinucleotides analyzed in this study.

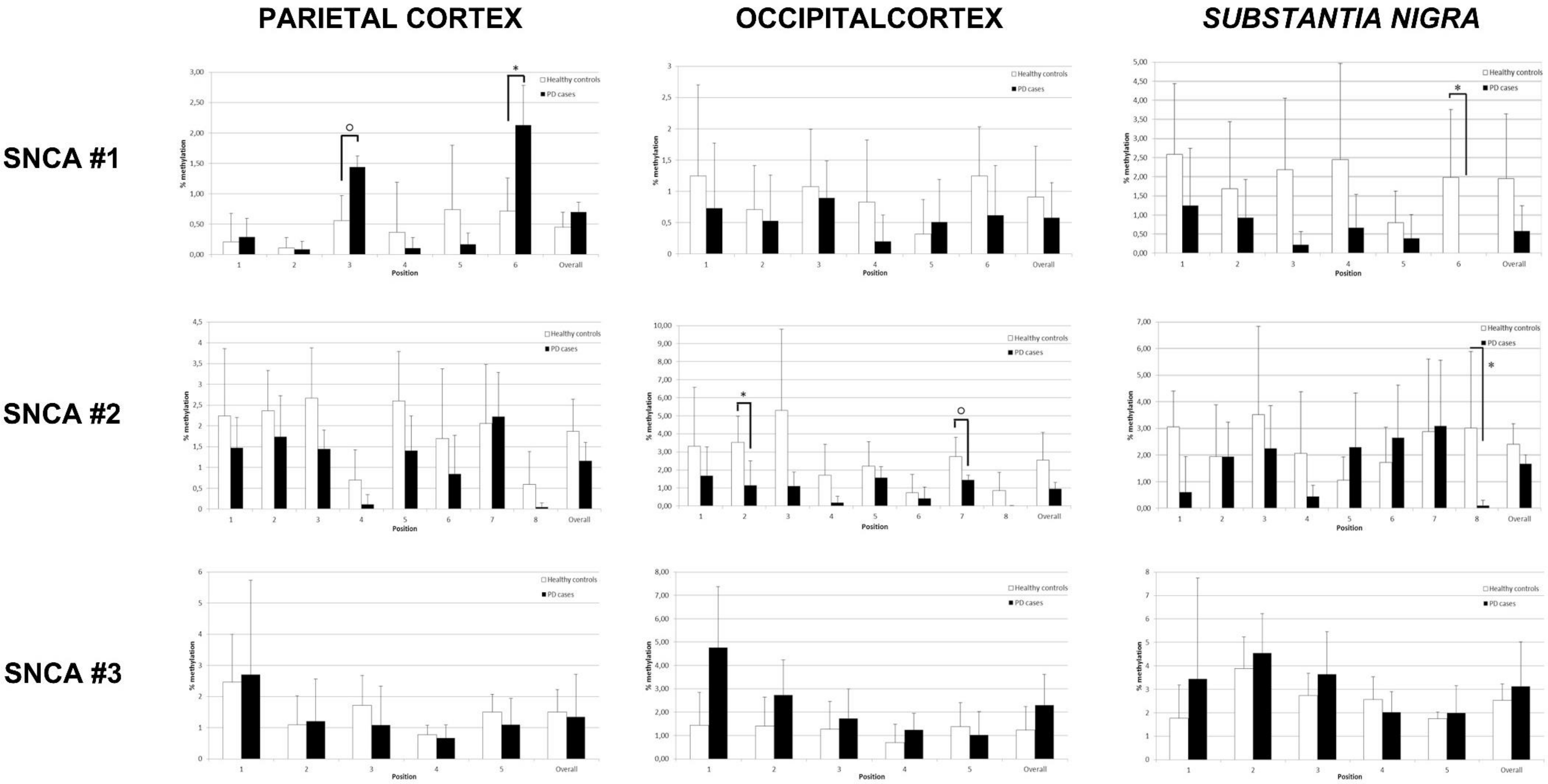

Figure S2. Methylation levels for the three assays in *SNCA* in the parietal and occipital cortices and *Substantia nigra* from PD patients and controls. The percentatge of methylated C for each CpG pair in the assays is shown (bars) together with its corresponding Standard deviation for PD patients (Black bars; n=5) and healthy controls (white bars; n=5 in parietal and occipital cortices, n=4 in *Substantia nigra*). The global level of methylation for each CpG island is also included (Overall). * $p<0.05$; º $p<0.01$

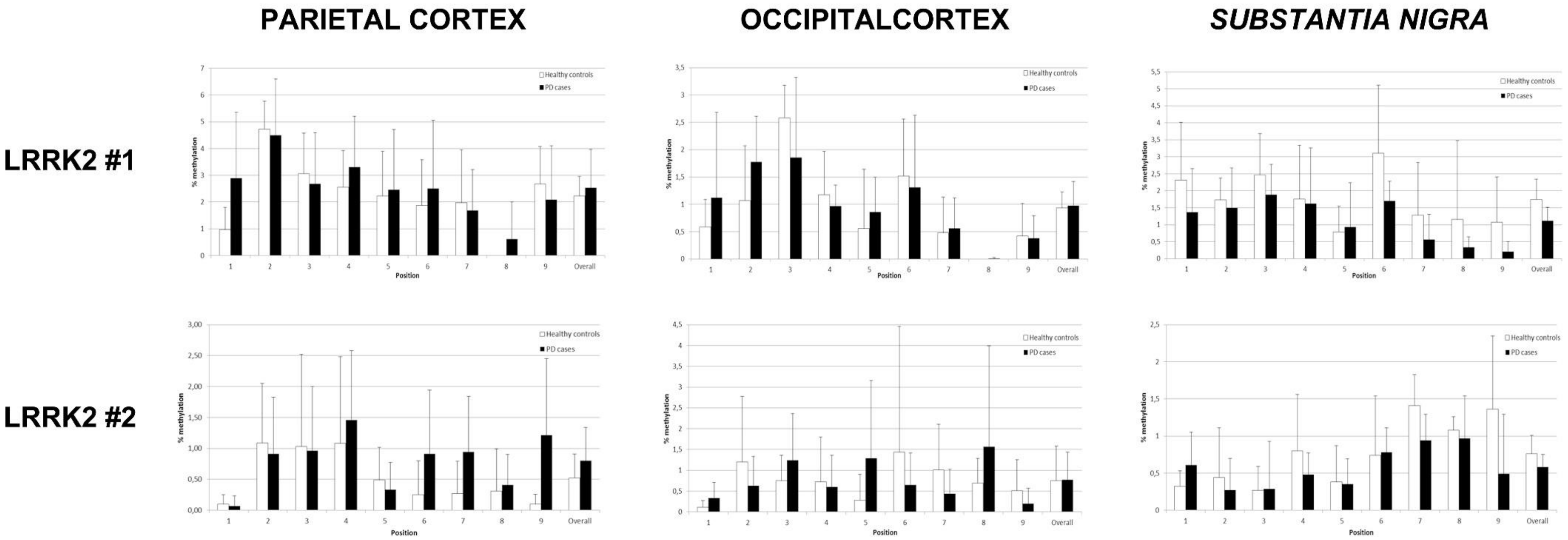

Figure S3. Methylation levels for the two assays in *LRRK2* in the parietal and occipital cortices and *Substantia nigra* from PD patients and controls. The percentatge of methylated C for each CpG pair in the assays is shown (bars) together with its corresponding Standard deviation for PD patients (Black bars; n=5) and healthy controls (white bars; n=5 in parietal and occipital cortices, n=4 in *Substantia nigra*). The global level of methylation for each CpG island is also included (Overall).

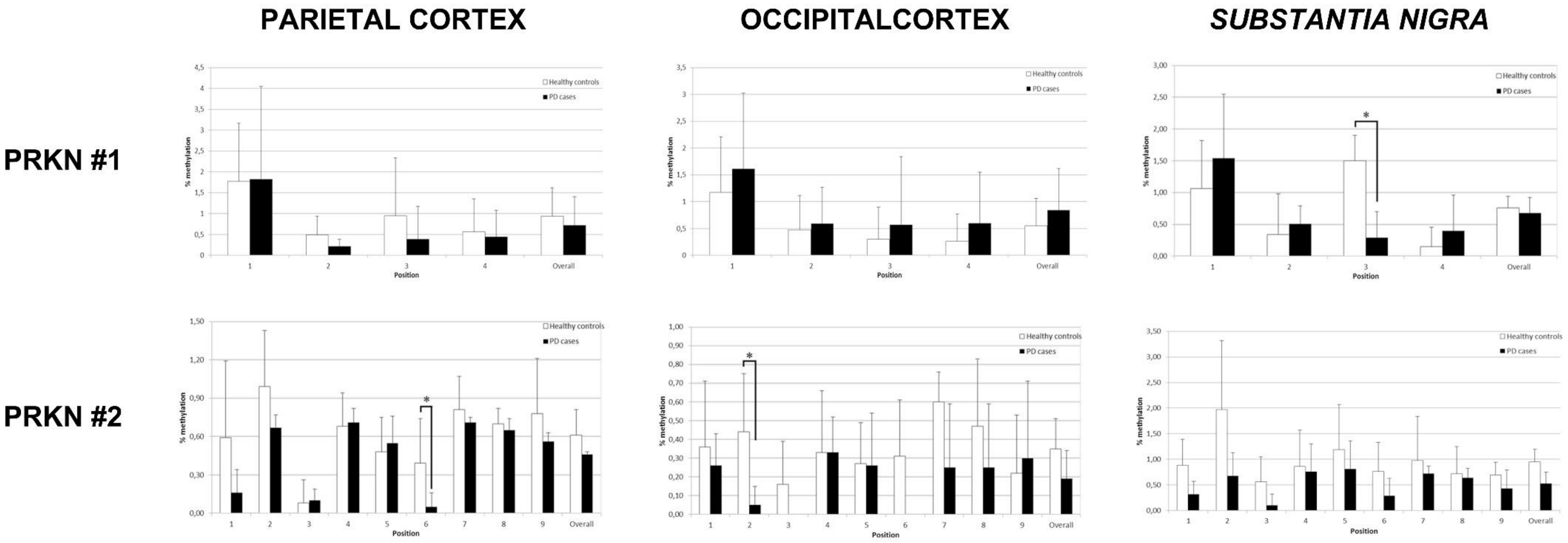

Figure S4. Methylation levels for the two assays in *PRKN* in the parietal and occipital cortices and *Substantia nigra* from PD patients and controls. The percentatge of methylated C for each CpG pair in the assays is shown (bars) together with its corresponding Standard deviation for PD patients (Black bars; n=5) and healthy controls (white bars; n=5 in parietal and occipital cortices, n=4 in *Substantia nigra*). The global level of methylation for each CpG island is also included (Overall). * $p<0.05$

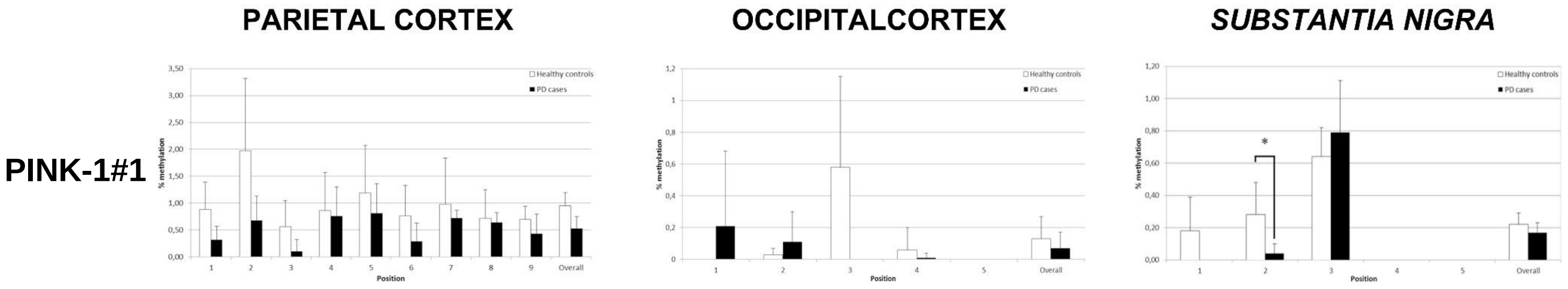

Figure S5. Methylation levels in *PINK-1* in the parietal and occipital cortices and *Substantia nigra* from PD patients and controls. The percentatge of methylated C for each CpG pair in the assays is shown (bars) together with its corresponding Standard deviation for PD patients (Black bars; n=5) and healthy controls (white bars; n=5 in parietal and occipital cortices, n=4 in *Substantia nigra*). The global level of methylation for each CpG island is also included (Overall). * $p<0.05$

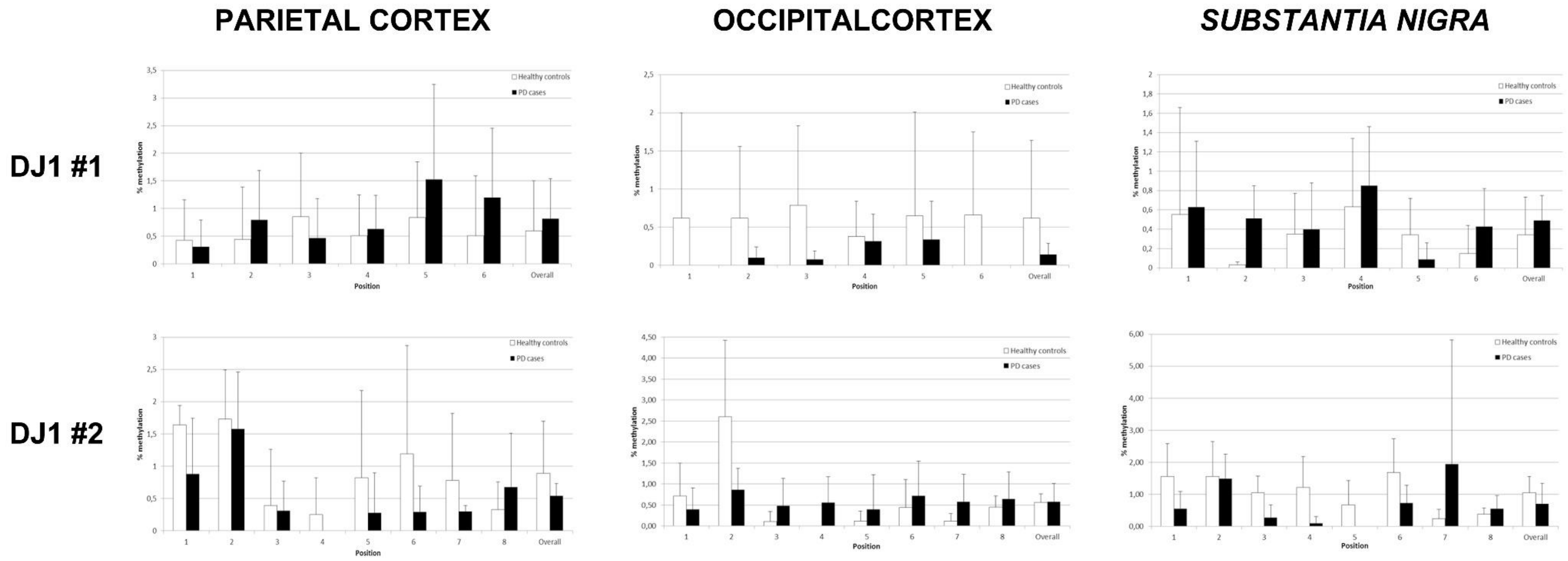

Figure S6. Methylation levels for the two assays in *DJ-1* in the parietal and occipital cortices and *Substantia nigra* from PD patients and controls. The percentatge of methylated C for each CpG pair in the assays is shown (bars) together with its corresponding Standard deviation for PD patients (Black bars; n=5) and healthy controls (white bars; n=5 in parietal and occipital cortices, n=4 in *Substantia nigra*). The global level of methylation for each CpG island is also included (Overall).
